# Old Physic and Young Physic; in School and out of School

**Published:** 1848-06

**Authors:** Wm. Treat


### BUFFALO MEDICAL JOURNAL

AND

### MONTHLY REVIEW.

VOL. 4. JUNE, 1848. NO. 1.

### ORIGINAL COMMUNICATIONS.

ART. I.—*Old Physic and Young Physic; in School and out of School.* By Wm. Treat, M. D.

[The following address was delivered before the Young Men's Association of this city. Although written for a popular auditory, it is believed that it will prove acceptable to the medical reader. It has been obligingly furnished for publication by request of the editor, which, it may be added, was made at the suggestion of several medical brethren who listened to its public recital.

The length of the address has rendered it necessary to exercise the editorial prerogative of marking out some portions, not inferior in excellence to that which is retained, and notwithstanding this curtailment, it is deemed advisable to divide it between two numbers of the Journal, so that it may not encroach on subjects of a more practical character.—Ed.]

The uncertainty of health—the uncertainty of life—the uncertainty of the weather—among the uncertainties, are the most fruitful, and, yet, often, most fruitless topics of conversation.

Our greetings, and our modes of salutation, in private and in the market place, appear to take their form from the uncertainties we have named. Here the opinions and suggestions for health, and probabilities of weather, of all our charities are most freely bestowed; often, we apprehend, for this reason—they cost us nothing.

To be able to foretell, to prognosticate the weather, one may obtain not a little insight by personal observation, and an accumulated personal experience, limited to the narrow range of one's own sentient organs, it is true, but such observations collected from the many, furnish the elements for basis upon which is upreared the science of meteorology, the province of which is to determine and define the unchanging laws which govern the changing wind and weather, subjects about which we are inclined to converse so much, knowing, as we do, so little.

Eighteen centuries ago, one who "spake as never man spake," said of the wind:—"It bloweth where it listeth, and no man hath knowledge whence it cometh or whither it goeth," and cited the then existing proverb "ye look upon the sky in the morning when it is red, and ye say we shall have rain, and it is so." The chain of causes traceable from these phenomena fall within the province of the natural philosopher; and a previous knowledge of the laws of light, reflected and refracted, must be presupposed, before the exercise of our reasoning upon them can be of much avail. Hence, scientific explorers have sought instruments of admeasurement, and joined to them, a technical lore as an instrument for conveying thought to others—compassing the whole range of natural phenomena, and comprising the observation of the *facts* collected from all observers—whereon they build their theories—their philosophy of the weather. In the exercise of their cabala, we hear certain magic words, solemnly pronounced, such as cumuli, strati, cirrrhus and cerro-strati, of barometers, thermometers, rosometers, pluviameters, and hygrometers—isothermal, irotheral, isochemical, and other lines, accompanied with mathematical calculations of physical counteracting agencies, such as mountains and valleys, oceans, lakes and gulf-streams, and, behold! we have mapped in outlines, the cycle of atmospheric changes, wanting a few minor elements to mark them with the same accuracy as the meridian lines upon an artificial globe. Results astounding to the uninitiated,—unheeded, uncared for, are such rules, and such demonstrable laws, by the isolated observer with whom we have just met on change, made our salutory inquiry, received the benefit of his experience, awarded the epithet of an old sea-dog, by consequence as weather-wise or weather-worn, and jostled on to our respective avocations basing thereupon the exposure of fleets upon our lakes or oceans, laughing in the pocket at our cheaply obtained opinion, and at the new-fangled instrument, a barometer, feeling quite assured it can foretell comparatively nothing, when put in requisition after the oracular opinion of this experienced son of Neptune!

Having by transposition made *our* salutary greeting, first upon the weather, we come now to speak of that other popular theme—individual health and that of our households. As to the public health, we must leave that to the multitude of political doctors, with whom, of course it is to be supposed there are none but knowing ones—here can be no quacks!

Since, with the many, our streets, our market places, and our social circles have been converted into forums for the discussion of elementary and profound science, and every new dogma, not to say absurdity, has there found its adherents, pertinaciously urging the merits of their utopiad, much need is there of the labors of the critic, with his fan ever in his hand to winnow out the few grains of truth. Especially has this become needful in this supposed age of innovation in the theory and practice of medicine.

In this departmant we design to speak of some of the most vaunted, and, at the same time, endeavor to refer them back to their lost parentage. As a preparative therefor, we are impelled to ask your indulgence, while, as concisely as possible, we trace the early history of medicine, and from their numerous schools indicate their parallels; in one discourse we can hardly hope to do more than this. For, unless schooled in the past history, one may well suppose never was there, like this present, such an accumulation of theoretic and experimental wisdom.

We venture nothing in affirming that observations made upon the phenomena of disease, and the operative action of medicine, have been accumulating from that period, when sin

"Brought death into the world and all our woe."

These observations, in proportion to the industry, facility, sagacity, and judgment employed for their accumulation, have been seized upon, gently swayed, or dragooned into hypothetic, or theoretic views of disease, and curative agency. Out of these have sprung the schools of medicine, holding each their own set of dogmas, and having their followers as devoted as those of modern party or sect, while in all time, including this present, there have been found solitary individuals laboring zealously to detract from the former to make way for a fabric of their own, and often with success, foisting upon the community and age dogmas absurd past ridiculous, and far beyond what the culture of their time would warrant. From their multiplicity we shall be unable to allude to many of these, though they would be found of service in elucidating our subject in its modern, or new school aspect.

There is a prevalent opinion that what is now termed regular practice in medicine has stood unassailed until the present period, and, now assailed, it must needs fall. The falsity of this will become apparent as we progress.

In the early history of our race, indeed for some centuries, we have reason to believe, there were no individuals or class of men, who devoted their lives to the acquirement and practice of the healing art. Division of labor, and singleness of occupation, which has wrought wonders in the whole range of physic and mental employment of men, is a more modern device. The art of prescribing for disease was vested in the multitude, and they expressed their opinions, and made their remedial suggestions, as opportunity presented, and a common sympathy, or enlightened benevolence dictated. The sick, even as late as our Savior's time, often sat in the gateway of some walled city, or other public place, in expectation of counsel from some passer by, who had become skilled by observation at home, or schooled by travel abroad. Indeed, among the Hebrews, law required such advice to be given. Yet, the sick were not left solely to such casual resources. To the priesthood was committed the especial care of the sick among the Jews. Indeed, the priesthood, in all time, and of all countries, have been the conservators of learning, to whom the world of literature is greatly indebted for the preservation of its sacred and profane lore.

Ante-dating the period of Jewish organized priesthood, the Egyptians had made respectable acquirements in the healing art, acquirements which they retained centuries after the Jewish nation or tribes were dispersed. Though, with the valuable, they associated the practice of magical incantations.

But it is from the final subjugators of the Jews—the Romans, and the crowned or rising empires of that period, we are to seek historical record of the exodus, the coming out from the learned a few individuals, though not Roman by birth, whose especial province was the culture and practice of the *art* of medicine—not then a science.

Much that is fabulous, no doubt, is interwoven in the biography of these persons and their times, augmented by tradition, superstitious reverence, or cunning device artfully wielded, and turned to the emolument of the avaricious, or fame of the aspiring would-be hero of the coming centuries. Indeed, so much of the fable find critical writers upon the history of this period, and of medical history, that attempts at the throne, and doubts of the personal existence, of one considered for ages the great founder of the medical art, have been, and by some, are even now entertained. By these, the stories of Æsculapias, and his temples, are placed in category with that part of Roman history which treats of Romulus, his foster mother the wolf, and his exploits while in that nursery. What this latter story is to Roman history, is that of Æsculapius to the history of medicine, neither the one nor the other can begin, much less advance, unless by conceding as verities much that is fabulous, as a spinning point, whereto may be attached the remote end of the thread of history.

In about the 13th century before Christ, we are told that Chiron, a prince, probably from Thessaly, became the instructor of a foundling reared by a shepherd, whose pupil made such proficiency in the medical art, that the jealousy of Pluto led to his death, because he rescued so many from the grave. In the person of this foundling we have the world-renowned Æsculapius. He transmitted his profession to his two sons.

Fom that great Pope, popular credulity, we must ask a special dispensation, in the outset, for entertaining belief in one hero and demi-god in the old school aspect of our theme, trusting we shall not ask in vain, when its liberality is considered in granting to what is termed the new school, one for almost every day in the calender. That a great man, full of practical wisdom, had being in the remote past, bearing some name, be it Æsculapias or some other, who wrought upon the imagination, as well as cured disease by potent remedies, we have no doubt,—one who bears in medical mythology the same relation that Odin, or Woden, does to the old Norse's ruling god among his chief divinities.

For 13 centuries and upwards, before the Christian era, temples dedicated to him were served by his reputed descendants, acting priest and physician in one person. These individuals were called Asclepiades. Though they, or their patients when cured, kept record of diseases treated, and medicines given, and deposited them in the temples, yet we have no record that from their protracted experience and observation the science of medicine made any progress in their hands up to the time of Pythagoras, about the 5th century, when Hippocrates, an Asclepiad, of the 18th generation from this demi-god, appeared, and officiated in the temple at Cos. He, as also the others in this temple, attempted an analysis of the facts in their collection, and based theories upon them ; and thus arose the Hippocratic or Rationalist school, in contradistinction to those priest-physicians at the temple of Gnidos, who, in their treatment of diseases, professed to disregard all theory, and rely solely upon collated facts — these latter were called Empirics.

These rival schools, or sects, were nearly balanced in numbers, for several centuries, and engaged in a war of words — since it is evident to us the one cannot reason without facts, and the other could do nothing with facts without an exercise of reason. The question with them, therefore, appears to resolve itself into a degree or governing proportion, which the one mode should have over the other.

The prevalent philosophy at the time of Hippocrates, (the Pythagorean) is visible in all his writings, and lies at the bottom of all his medical hypotheses. Up to this day, and so it ever must be, medicine, as a science, can only keep pace, and never move in advance of the current philosophy, or hand maiden sciences which minister to it. Like Geology, its birth and advances are chained by an inevitable necessity to the sister sciences, and, as a consequent, its car must move in rear, or in train with them, or ever remain stationary.

Asclepiades of Bythnia, who flourished in the time of Pompey, 50 years B. C., was a follower of Epicurus in his philosophy, adopting his doctrine of atoms and pores, (while he practiced medicine as a mere charlatan.) He advanced a new theory, on the supposition that all diseases arise from the obstruction of the pores, and irregular distribution of the atoms, of note as a leading feature with a modern sect, who practice steaming thus to open the pores.

His pupil, Themison, founded the Methodic school, which flourished the first two centuries after the Christian era, and out of it uprose, as subdivisions, the Pneumatic, from *pneuma,* spirits, which were believed, with solids and fluids, to compose the corporeal frame, and the Eclectics, of no note in this connection.

At this period Galen appeared. Schooled by travel in the learning of the age, he was invited to remove from his native place, Pergamus, in Lesser Asia, to Rome, by the Emperor Aurelius. The weight of his name thirteen centuries did not suffice to overcome, and in all this time his opinions were deemed a sufficient argument against any new hypothesis, or alleged matter of fact.

The science of medicine withstood the shock, longer than science and literature in general, during *the middle ages,* but with these finally retreated to the Moors and Arabians, who were preservers of the works, and imitators of Galen. By the intervention of the Crusaders it was brought into Italy and France, having gathered in Arabia a new feature from the science of Chemistry, then in its inception. Out of this arose the sect of Chemists, and a contest with the Galenists followed, which continued through the 16th century. Indeed, there comes, booming over the night of time, its echo, yet heard in cry of nominal botanists against the mineral doctor! A cry, at this late day, as senseless as one from the awakened century sleepers would be against witchcraft, such have been the changes in medical science and medical practice! The Galenists were *then,* for the most part, more learned and scientific than their adversaries; they counted in their ranks the University professors (which institutions were planted all over Europe in period from 13th to 16th centuries.) Their pupils, although strongly attached to Galen, were, in some degree, independent observers of the phenomena of disease, and collators of facts. Yet, their practice was complicated, and inert ; their medicines were derived principally from the vegetable kingdom (though the animal was made tributary.) Their prescriptions were long and multifarious, consisting of a large number of ingredients, combined together in such a manner as to render it almost impossible to conceive the probable operation of the compound.

The *Theriaca Andromachi* is an example. This contained, with flesh of vipers, sixty-two vegetables, and was used as a counter poison for more than 16 centuries. The inventor of this villainous compound was the Archiater, or royal physician to the Emperor Nero, and we find it approved by Manget, royal physician to Frederick I, king of Prussia, as late as 1703, from whom we shall quote hereafter. Each ingredient was believed to have the properties, thus combined, of effecting a specific cure upon some one disease — thus exceeding a vaunted remedy of our city, which boasts of 22 vegetable remedies, so happily combined, as to work wonders in this our day.

The *Chemists* were bold empirics at first, but, in time, acquired skill and science. They discarded the long prescriptions of the Galenists, rejecting a great many of the articles of their pharmacopæia, while they introduced the active metallic preparations, and made free use of most powerful articles of all kinds. Thus both were reprehensible — the Galenists for use of weak, and often inert agents ; while the Chemists were rash in the use of potent ones.

As we have some old, as also modern authors, who were Galenists before us, we have thought it might be of interest to give a few examples from each of these, illustrative of Galenic practice, for centuries ; while, at the same time, they serve as answers for many modern sects, whose disciples boast of experience, and their personal observation of facts, to the accuracy of which they are ever ready to vouch, as well as to cite authority, for the like, from some of the rulers of the land, as well informed perhaps, as themselves.

In an astrological Herbal written by Nicholas Culpepper, the son of a puritan clergyman, practicing physician two centuries ago, though not of the College, but popular in his day, we find every plant is by catalogue enumerated, and the planet which governs it named. Here we read of:—

" *Onions.*—Mars owns them, and they have gotten this equality to draw any corruption to them, for if you peel one, and lay him upon a dung hill, you shall find him rotten in half a day, by drawing putrefaction to it. Thus being bruised and applied to a Plague sore, 'tis very probable 'twill do the like!" Sundry modes of preparation are then given, not differing materially from any modern matron, who has the practice from tradition.

Of *Saffron*, and the oft met-with saffron tea in the nursery, we have the following cogent reason for the continued use of an inert plant:—" It is an herb of the sun, (judged by its yellow color,) and therefore you need not demand a reason why it strengthens the heart so exceedingly. Let not above ten grains be given at a time, for if the sun, which is the fountain of light, may dazzle the eyes, and make them blind, a cordial, taken immoderately, may hurt the heart, instead of helping it. It quickeneth the brain, for the sun is exalted in Aries, as well as he hath his house in Leo. It is excellent in epidemic diseases, such as measles, and is a notable remedy for yellow jaundice." So saith also any coterie of old women, and we know that children often cry for it, as is advertised of certain lozenges, for reason to be found, we apprehend, in the vigorous hand which plies the often repeated and well filled spoon.

Of *Golden rod*,—He says:—" Venus claims the herb, and therefore, to be sure, it restores beauty lost." Not unlikely, in this Golden age, if they were ingots of gold.

*Tansy*, which, he assures us, is woman's best companion, their husband excepted—though its virtues are unlike the

*Periwinkle*, which, eaten by man and wife together, causes love between them.

And the sighing Benedict may find help for *envy* and *despair* in that quarter, by drinking a decoction of *Melancholy Thistle*, which expels superfluous melancholy besides, and makes a man as merry as a cricket. Diascorides saith the root borne about one doth the like. Modern writers laugh at him: *Let them laugh that win.* My opinion is, that it is the best remedy against melancholy diseases that grows. They that please may use it.

Of *Rue*, our author says the leaves mixed with figs, walnuts and juniper berries, forms Mithridates' counter poison, (a mixture, by the bye, that has stood the test of centuries, and now is exploded,) is used against the plague and all venomous poisons are rendered harmless by it—*slanderers to the contrary*—" He that doth use but the quantity of a hazl nuet of that receipt every morning, shall preserve his body in health if he do but consider that Rue is an herb of the Sun and under Leo, and gather it and the rest accordingly!"

Shade of Sanctorius!—in this thy day of invention of the thermometer, thou didst announce thyself as author, and publish "On the method of avoiding errors in medicine." We apprehend from this, and what treatises we have yet to quote from a later age, thy book must have fallen cold as zero upon the leaden public—for the froth, by ebullition, which has followed since in medicine, indicates a boiling point somewhither. Is it in the first that follows. We will see;—

A book by the Rev. John Wesley, entitled "Primitive Physic, or an easy and natural method of curing most diseases," is now in our possesssion, the edition 26th. A valuable work one would think this, which has met with such public favor, and been subject to revision by the author from time to time, so arranged by notation of the best remedies for every disease named, and often with the additions of "Good," "have tried it," and an occasional *I.* denoting infallible! As at this present day the testimonials of distinguished clergymen in favor of new remedies in medicine are most valued, (though in our city, to *their* credit, not often to be procured,) we offer a few endorsed by this great leader, not to say founder of a religious sect. This we do the more freely by way of atonement for the hard names, deserved of course, which he bestows on the regular physician for selfishnesss in locking up the ripe knowledge and practice of medicine from the multitude. He evidently is no rationalist, but must be classed with the Empiric, as he denounces all theory, &c. We thumb his papers, and copy:—

"*For the Ague:* 5th best out of 28 remedies: apply to the stomach a red hot onion, slit. 6th best, frankincense and nutmeg spread on linen and applied to the pit of the stomach. I have never yet known it to fail."

"*For the Asthma:* 4th best out of 17 remedies: Live a fortnight on boiled carrots only. It seldom fails."

"*Bleeding at the nose.* No. 1, drink largely and eat much raisins."

"*A Cancer in the Breast*: Take warts that grow on the inside of horses' fore legs, dry them, powder, sift, and steep in two quarts of ale, drink half a pint every six hours in warm new milk. It has cured many. *Tried.*"

We have thus far only turned the first 20 pages of this book. A few more of these recipies from it and we have done with it.

"*For Hooping Cough.*—Rub the feet thoroughly with hog's lard, before the fire at going to bed, and keep the child warm therein. Tried."

"*For a Consumption:* Cold bathing has cured many deep consumptions —*tried.* Or, every morning cut up a little turf of fresh earth, and lying down, breathe into the hole for a quarter of an hour. I have known consumption cured thus."

"*For Gout in any Limb*: Rub the part with warm treacle, and then bind on a flannel smeared therewith. This has cured an inveterate Gout in 36 hours. Regard not them who say—The Gout ought not to be cured. They mean it *cannot.* I know it cannot by their *regular prescriptions.* But I have known it cured in many cases without any ill effects following. I have cured myself several times. Treacle in same way cures rheumatism. Tried.

"*For Scrofula, or Kings Evil:* Drink half a pint of tea twice a day, for four months, made from a dried burdock leaf. I have known this to cure hundreds."

"*For a broken shin:* Bind a dry oak leaf upon it, or put on a bit of white paper moistened with spittle. *Tried.*

"But fasting spittle, outwardly applied every morning, has sometimes relieved and sometimes cured Blindness, deafness, contracted sinews, corns, warts, &c.

Taken inwardly, it relieves, or cures Asthmas, cancers, epilepsy, gout, king's evil, leprosy, palsy, rheumatism, scurvy, stone, and swelled liver.

"The best way is, to eat about an ounce of hard bread, every morning, fasting two or three hours aftr. This should be done in stubborn cases, for a month or six weeks!"

In short this book is the mother of abominations, and some one of its infallibles has to be met and answered daily in the physician's round. Its eye washes must have destroyed the sight of thousands!!!

Of Galenism, its ancient and modern advocate, these examples may well suffice. In compounding the witches cauldron, Shakspeare made no draft upon his imagination, but wrote of popular remedies in his day — of the animal part of the contents of the cauldron, we shall give examples speedily from one or more writers in about the beginning of the 18th century, but now leave Galenists with their

"Roots of hemlock, digg'd i' the dark
And slips of yew
Shiver'd in the moon's eclipse,"

to which is added "grease that's sweaten from a murderer's gibbet," and jump our history of medicine with a brief summary of its present condition evolved from the old schools :

A multiplicity of rival schools and teachers throughout Europe, begat their legions of theorists, as well as practical observers, and, as in preceding instances, cited more in detail, they all contributed, and are contributing to the advancement of the science of medicine. From this eminence its votaries overlook hill and dale in the past periods of medical history, holding no quarrel with the disciples of its former schools, but rest content with culling from their practical wisdom ; viewing nought of too little import to be passed unheeded, in its comprehensive grasp seizing all, and submitting them to the crucible of a searching scrutiny.

Its chief element is *Hippocratic*, or Rational, in that it demands the exercise of a sound judgment — *Empiric*, in quest of facts throughout all the various schools, conjoined with personal observation — *Galenic*, in search of vegetable medicaments — yet *Chemical*, in not discarding minerals deemed really valuable. *Anatomical*, in that it would know of the mechanism of the body in health, in order to treat it when in a morbid state — *Physiological*, in that it seeks a knowledge of the uses of the various parts of the body, that a perverted action may be known, and ministered to—*Mathematical*, as well as of *Vitalists*, in so far as pneumatic, hydraulic, and muscular forces, are found in common contributing to the movements of fluids and solids of the body — and *metaphysical*, in that it recognizes the action of mind upon matter as a curative agent— and these all combined constitute, what now it really is in name and teaching, the *Eclectic school.*

With these elements from the various schools, and many more not enumerated found in it, wherein lies the foundation to the accusation that the old school, or Eclectic, is not open to improvement, but remains ever incrusted in its views :— unless it be incrusted in an inflexible conservatism, always penetrable to truths which presents in harmony with the sciences and philosophy of the age ? In passing hastily over the dogmas which constitute its elements, we are not disconcerted at the charge of incrustation, but accept it, when we take into view the habits and growth of the animated crustacea, even as presented in the largest of the tribe, *the lobster,*— of which we are told, in the growth by assimilation of the food of life, for want of room he bursts his crusty shell — expands nearly one third in size, and, by secretion, forms another, and, by repletion, yet another shell to be cast as before. Is not here the evidence of growth ?

But naturalists inform us there is another species of lobster, the soldier, or lobster crab, that comes from, we know not where, but appears from over-land, at the ocean side, when the true lobster sheds his crusty shell·

But what Nature has denied this animal, it takes care to supply by art: and taking possession of the shell of some other animal, it resides in it, till, by its growth, it is compelled to seek another. Hear what a celebrated Naturalist says of him: —

"The little soldier is seen busily parading the shore along that line of pebbles and shells which is formed by the extremest wave, still, however, dragging its old incommodious habitation, [like the traditionist] at its tail, unwilling to part with one shell, even though a troublesome appendage, till it can find one more convenient. It is seen examining one after the other, occasionally slipping its tail from the old habitation to try on the new, till, at last, it finds one light, roomy and commodious; to this it adheres, though the shell be sometimes so large as to hide the body of the animal, claws and all, and then it retreats to the mountains again."— *Buffon, etc.* 18, 210.

Having devoted more time than we intended to the growth of the old, or Eclectic school, we hope to compensate for it by the facility it promises to afford by way of elucidating the *New;* cast off shells, *membrana disjecta*, or fragments, constituting, with some of them, their sole elements.

*Thomsonianism* or the Botanic school, as it delights to be called, (though we never had the good fortune to meet with a member of it who had knowledge of botany) we have seen cleaving to Galenism, and its body hid in it, and decrying Chemicals, while their caudal end is, at the same time, fixed to the doctrine of obstructed pores which preceded it — there we must leave it — with help of trumpet of fame of a Wesley, and Culpepper, to bear its cry into the present century.

In compliance with the announcement of this lecture we come now to the task of condensing a narrative of some of the most imposing pretenders in medicine *out* of school. Had we time we might be disposed to set these forth in order of time, or notoriety, but now must be content to present them, as, like the ghosts of the past, they flit before the memory, and again sink to the deep profound with goblins damned; for

——— "many shapes
Hath Death,—and many are the ways that lead
To his grim cave."

Of specialities, working cures, their name is legion. Look into any newspaper, even those called religious, and you may read of them. The public treasury in Great Britain, and France, has been levied upon largely, almost yearly, to purchase the secret, or reward some nostrum vender, each differing from, and giving way to another, and all having yet one common characteristic, worthlessness! The *eau de Medicinale*, for Gout and Rheumatism; Madame Nouffer's medicine for Tape and other worms, were of this character.

The biography of one St. John Long, a basket-maker, in Ireland, elevated to post of gentleman's servant, speaks of their success. *Long* invented a Liniment, which netted £20,000 a year, and not only was thus elevated by wealth from an humble station, but many branches of his family also. His liniment has long since ceased to be efficacious! David Hartley, a physician of last century, was instrumental in procuring a reward from the British Parliament for Mrs. Steven's Lithontriptic, the chief ingredient of which was soap. Hartley took of this nostrum, and died, it is said, after having taken, in all, 200 pounds of soap!

Sir Robert Walpole, prime minister to George I and II of England, also died a victim to the use of a Lithontriptic.

But these illustrative materials accumulate at our hands. We give place for an account of an Elixir of Life, of American origin, as related by a Lord spiritual, Bishop Berkeley, of whom a biographical dictionary, now before us, published in Hartford, Conn. in 1845, says:—"The excellence of his moral character is conspicuous in his writings; his philosophical discoveries, particularly of the medical virtues of a medicine which we shall soon name, were of great service to mankind. He certainly was a very amiable, as well as a very great man, and Pope is scarcely thought to have said too much when he ascribed to

"Berkeley every virtue under Heaven."

Hear his testimony:—"I am indispensably obliged by the duty I owe to mankind to make my experience public!

This precious fluid is made by stirring a gallon of water with a quart of tar, leaving it 48 hours, and pouring off the clear liquor. It is a preventive of small pox, a cure for impurities of the blood, coughs, pleurisies, peripneumony, erysipelas, asthma, indigestion, cachexia, hysterics, dropsy, mortification, scurvy, and hypochondria. It is also of great use in gout and fevers, and an excellent preservative of the teeth and gums, particularly is it beneficial to sea-faring persons, ladies, and men of studious and sedentary lives."

The Bishop is apprehensive he may be suspected of reading from a quack advertisement. He proceeds:—

"From my representing it as good for so many things, some may perhaps conclude it is good for nothing. But charity obligeth me to say what I know, and what I think, however it may be taken. Men may censure and object as they please, but I appeal *to time and experiment.* Effects misimputed, cases wrong told, circumstances overlooked, perhaps, too, prejudices and partialities against truth, may for a time prevail, and keep her at the bottom of the well, from whence, nevertheless, she emergeth sooner or later, and strikes the eyes of all who do not keep them shut."

The wonder-working power of the *Royal-touch* we must trust the graphic pen of Shakspeare to set forth.

MALCOM asks : Comes the king forth,
I pray you?
DOCTOR. Ay, sir : there are a crew of wretched souls,
That stay his cure—their malady overcomes
The great assay of art; but at his touch,
Such sanctity hath heaven given his hand,
They presently amend.
MACDUFF. What's the disease he means ?
MALCOM. 'Tis called the evil :
A most miraculous work in this good king :
Which often since my here remain in England,
I have seen him do. How he solicits heaven,
Himself best knows : but strangely visited people,
All swollen and ulcerous, pitiful to the eye,
The mere despair of surgery, he cures :
Hanging a golden stamp about their necks,
Put on with holy prayers : and 'tis spoken,
To the succeeding royalty he leaves
The healing benediction. With this strange virtue,
He hath a heavenly gift of prophecy.—*Macbeth, IV.* 3.

The healing benediction has passed from royalty now, and the evil of scrofula has, by a strange vicissitude, taken its place, and the beggar, if he be the seventh son of a seventh son, is born to, and now wears this healing mantle of the prophet.

Of professed Indian doctors, professed learners of pow-wows or meta men, their name is legion, yet it is well known to the profession that our aborigines had no skill in medicine, save in the use of a few simples. Dr. Rush, of Philadelphia, a man who needs no better voucher for his fidelity than that name which he affixed to the U. S. Constitution, gathered what was known of them in his time. The medical corps acting as agents for our government in Indian affairs in the far west, have sought zealously for this reputed skill, but all report it to be idle rumor. What the Indian's skill, may be learned in any work on the early history of this country, say Bancroft's. We are informed of Gov. Winthrop's journey to cure Massasoit—"The priests, or pow-wows, were their physicians, who practiced for the relief of the sick, magical ceremonies and incantations. They acted in the character of witches, calling on the Devil to assist them * * promising sacrifices of the best things they possessed to the fiend, if he would come to their help. A pow-wow could not work his witchcraft in the presence, nor would they have any effect upon the persons of the English—like mesmerism, first of all, it required implicit faith. We question if any of our boasted Indian doctors have been duly instructed after their mode. Here follows the schooling :

"Young men who were educated for pow-wows were forced to swallow nauseous draughts for an emetic, and when the contents of the stomach were thrown up, they were obliged to swallow the same, again and again, until the stomach itself was almost inverted."

We would as soon rely upon that sovereign specific at Tassura, in Egypt, "Koran water," made from leaves of the Koran, as upon Indian specifics at such hands.

German doctors without vouchers as to their education, are as little to be relied upon, the charlatans among them, having learned by tradition, follow the teaching of Manget, and use the most offensive animal substances, to this day kept by apothecaries in that country. Some of these we shall anon enumerate.

(*To be concluded in our next No.*)

